# Commissioning through competition and cooperation in the English NHS under the Health and Social Care Act 2012: evidence from a qualitative study of four clinical commissioning groups

**DOI:** 10.1136/bmjopen-2016-011745

**Published:** 2017-02-09

**Authors:** Pauline Allen, Dorota Osipovič, Elizabeth Shepherd, Anna Coleman, Neil Perkins, Emma Garnett, Lorraine Williams

**Affiliations:** 1 Department of Health Services Research and Policy, London School of Hygiene and Tropical Medicine, London, UK; 2 Centre for Primary Care, Institute of Population Health, University of Manchester, Manchester, UK

## Abstract

**Objective:**

The Health and Social Care Act 2012 (‘HSCA 2012’) introduced a new, statutory, form of regulation of competition into the National Health Service (NHS), while at the same time recognising that cooperation was necessary. NHS England's policy document, The Five Year Forward View (‘5YFV’) of 2014 placed less emphasis on competition without altering the legislation. We explored how commissioners and providers understand the complex regulatory framework, and how they behave in relation to competition and cooperation.

**Design:**

We carried out detailed case studies in four clinical commissioning groups, using interviews and documentary analysis to explore the commissioners’ and providers’ understanding and experience of competition and cooperation.

**Setting/participants:**

We conducted 42 interviews with senior managers in commissioning organisations and senior managers in NHS and independent provider organisations (acute and community services).

**Results:**

Neither commissioners nor providers fully understand the regulatory regime in respect of competition in the NHS, and have not found that the regulatory authorities have provided adequate guidance. Despite the HSCA 2012 promoting competition, commissioners chose mainly to use collaborative strategies to effect major service reconfigurations, which is endorsed as a suitable approach by providers. Nevertheless, commissioners are using competitive tendering in respect of more peripheral services in order to improve quality of care and value for money.

**Conclusions:**

Commissioners regard the use of competition and cooperation as appropriate in the NHS currently, although collaborative strategies appear more helpful in respect of large-scale changes. However, the current regulatory framework contained in the HSCA 2012, particularly since the publication of the 5YFV, is not clear. Better guidance should be issued by the regulatory authorities.

**Strengths and limitations of this study**

- This qualitative study provides unique, contextually rich insight into the views on and the use of competition and cooperation in commissioning in four clinical commissioning groups in England between 2013 and 2015.
- This study uses only four in-depth case studies, so it may not be representative of all national developments.
- As routinely collected data about the use of competition (or cooperation) in the National Health Service (NHS) are not currently collated centrally, the study could not ascertain the extent and nature of the use of competitive commissioning in the English NHS.

## Introduction to NHS quasi-market and competition policy

This paper reports a recent study of the use of competition and cooperation by commissioners in the NHS since the coming into force of the Health and Social Care Act 2012 (‘HSCA 2012’). In order to understand the context within which commissioners chose to use combinations of these two mechanisms to attempt to improve value for money and quality of services, it is necessary to understand recent developments in the structure of the NHS quasi-market in England, particularly as they relate to competition and its regulation.

The NHS was established initially in 1948 as a hierarchical public organisation. In the late 1980s, a quasi-market, incorporating competition between providers of care, was seen by the then Conservative government as the best form of governance structure for the NHS, which would improve value for money and quality of care.^1^ The New Labour government ‘went with the grain’,^2^ and retained the quasi-market structures, despite also focusing on top-down, hierarchical measures, such as performance targets.^3^ After the general election in 2001, there was an increased emphasis on markets and choice.^4–6^ The key measures were as follows: (1) *Demand side reform,* being enhanced patient choice which was designed to improve services. It was thought that patients would avoid underperforming hospitals, and the prospect of losing funding under the cost per case Payment by Results pricing system (see below) would create incentives to improve quality. (2) *Transactional reform*: a national tariff of fixed prices for procedures, known as ‘payment-by-results’ (PbR).^7^ Each episode of care reimbursed (or lost to another provider) would be charged at national tariff rates. PbR was initially designed to cover acute hospitals’ work, and it has not been expanded to community health services (CHS) or mental health services (MHS)—which still subject to block contracts, which amount to fixed budgets in effect.^8^

(3) *System management and regulation*: In addition to the continuing role of the hierarchical, top-down command and control system run from the Department of Health (and since 2013, NHS England) in the form of compulsory policies, the economic regulation of this system was undertaken by an arm's length body, the Cooperation and Competition Panel (CCP) which advised the Department of Health in accordance with the *Principles and Rules for Cooperation and Competition* (PRCC).^9^ The CCP's recommendations were not legally binding. The principles included the requirement for ‘providers and commissioners to cooperate to deliver seamless and sustainable care to patients’ (principle 4), while also prohibiting commissioners and providers from reaching ‘agreements which restrict commissioner or patient choice against patients’ or taxpayers’ interests’ (principle 6). At the same time, aspects of European procurement law applied to the NHS in respect of two main issues—procurement by the NHS had to be transparent and non-discriminatory between different types of providers. Some commentators believed that European competition law applied to the NHS during this period, but there were no cases to test this point.^10^ The other important regulator was (and is-see below for its enhanced powers) the independent regulator of Foundation Trusts (FTs) (see below) called Monitor.^11^ (4) *Supply side reform*: The first reform to the supply side under New Labour was the introduction of NHS FTs. While FTs are still owned by the state, they represent a more autonomous organisational form.^12–14^ Commissioners were also encouraged to engage with new providers from the ‘third sector’^15^ and for-profit providers were also encouraged to enter the NHS quasi-market on a larger scale.^16^

A further wide-ranging set of reforms was introduced into the NHS under the coalition government (2010–2015). The relevant aspects of these reforms were designed to increase the market-like behaviour of providers of care and they span the coalition government and current Conservative regime.^17^ The idea is the same as that behind previous versions of the NHS quasi-market: that competition between a wider range of providers will produce the desired results of improved quality and greater efficiency. The HSCA 2012 made a direct correlation between competitive behaviour in the NHS and competition law.^18^ The NHS Procurement, Choice and Competition Regulations No. 2 2013 (the ‘Procurement Regulations’) made under the HSCA 2012 include elements of existing guidance that were not previously subject to statutory regulation, including the PRCC and NHS procurement guidelines. The Procurement Regulations indicate that competitive procurement is to be preferred. Under the HSCA 2012, Monitor (as the economic regulator for the whole of the NHS) took over some of the functions of the former CCP and, along with the national competition authorities (being, since April 2014 the Competition and Markets Authority (CMA), and prior to that, The Office of Fair Trading, (OFT) and the Competition Commission (CC)), has powers to enforce competition law to prevent anti-competitive behaviour. At the same time, Monitor is also responsible for promoting cooperation. (HSCA 2012, section 66 (e)). As Davies^18^ points out, the provisions of the HSCA 2012 concerning competition are hard to follow and may be inconsistent. It is the role of NHS commissioners (including clinical commissioning groups ‘CCGs’—see following explanation), however, to ensure that the appropriate levels of competition and cooperation exist in their local health economies.^17^ The HSCA 2012 abolished the previous NHS commissioning organisations known as primary care trusts (PCTs) and replaced them with smaller (also) statutory organisations known as clinical commissioning groups (CCGs). CCGs are significantly different from PCTs because (as well as covering much smaller populations), general practitioners (GPs) were in control rather than NHS managers (with some GP representation) as was the case for PCTs. This change in commissioner organisation can be seen as continuing the English NHS's policy of surrogate planning, in which a negotiated order involving micro-commissioning, provider competition, financial incentives and penalties are the dominant media of commissioner power over providers.^19^

*The Five Year Forward View* (5YFV) issued by NHS England in 2014 did not mention competition between organisations and instead focused on how organisations in the NHS need to cooperate with each other, and in fact at times merge to form larger organisations. The 5YFV has been seen by commentators as an important indication of the direction of travel for organisational issues in the NHS.^20^ The Secretary of State for Health (Jeremy Hunt) has indicated that patient choice (ie, competition) may not be the best way to improve many services.^21^ On the other hand, Monitor argued that competition still had an important role in the NHS, despite the 5YFV.^22^ Importantly, it should be noted that there have been no relevant legislative changes, so the HSCA 2012 remains in force.

### Need for research on competition in the post-HSCA 2012 NHS

While studies have noted that incentives for competition and cooperation exist in healthcare, few have researched the interaction between the two.^23^ ^24^ At the same time, extensive research, based on transaction costs theory, demonstrates that markets are not always the most efficient institutional structures compared with more hierarchical forms, and this research extends to health services.^25–28^ Research indicates that the first Conservative quasi-market (1989–1997) was not entirely successful.^29^ ^30^ As Tuohy points out, the internal logic of the relevant system will affect the implementation of the policy change.^3^ Although state hierarchies can make abrupt strategic changes, they are vulnerable to problems of delay. In the case of the NHS quasi-market in the 1990s, the established logic of hierarchical corporatism blunted the effects of the market. Thus, the key to understanding the NHS quasi-market was (and is) its institutional context. Analysis of this demonstrates the persistence of the hierarchical nature of the NHS during the first Conservative government quasi-market period, as opposed to any marketised elements.^2^ Researchers found that the incentives to behave in market-like ways were not robust, and the hierarchical elements of the NHS under which the Department of Health was able to issue commands to the lower levels continued to hold sway.^2^ ^3^ ^30^

Furthermore, research studies about more recent incarnations of the NHS quasi-market demonstrate that it continues to contain very strong hierarchical elements, despite the increasing series of pro-market reforms since 2001.^8^ ^31^ It should be noted that there is research about the effects of competition in New Labour's version of the quasi-market, which finds in favour of competition in some acute services on efficiency and quality grounds.^32^ ^33^ Given the paucity of empirical research evidence in respect of the effects of the HSCA 2012, there is a need to investigate the way in which local health systems are managed to ensure that cooperative behaviour is appropriately coexisting with competition. (Although Krachler and Greer^34^ undertook a study of the relationship between marketisation and privatisation in the post HSCA 2012 NHS, this focused on market entry by private providers, rather than NHS commissioners’ behaviour to all providers).

An important aspect of understanding how competition and cooperation operate together is to investigate the rules which govern these interactions between people and organisations.^35^ In the case of the English NHS, these rules consist of legislation (primary, ie, acts of parliament; and secondary, ie, statutory instruments) and policies issued by the Department of Health and NHS England. Some specific forms of cooperation have been evaluated (such as integrated care^33^ and clinical networks), but the manner in which local health systems were being managed to balance competition and cooperation under the HSCA 2012 has not been investigated.

### Study of commissioning through competition and cooperation

In light of the absence of evidence about the operation of the HSCA 2012 from the view point of CCG commissioners, we undertook a study to investigate how commissioners in local health systems manage the interplay of competition and cooperation in their local health economies, looking at acute MHS and CHS. The research questions were:

- How do commissioners and the organisations they commission from understand the policy and regulatory environment, including incentives for competition and cooperation?
- In the current environment, which encourages competition and cooperation, how do commissioning organisations and providers approach their relationships with each other in order to undertake the planning and delivery of care for patients?
- How do commissioning organisations use or shape the local provider environment to secure high quality care for patients? This entails examining how CCGs’ commissioning strategies take account of the local configuration of providers and the degree to which they seek to use or enhance competition and/or encourage cooperation to improve services.

### Study design and methods

The study consisted of a series of four in-depth case studies to investigate how commissioners approached their roles as shapers of the local health system in respect of competition and cooperation issues.

The selection of a case study design for this research was appropriate for several reasons. The research questions were suited to a qualitative rather than quantitative approach, and case studies can accommodate these methods.^36^ Qualitative research is based on data generation methods which are flexible and in which categories are not fixed prior to research, but can alter in response to the data which are collected. Moreover, case study designs are thought to be particularly sensitive to exploring a ‘contemporary phenomenon within its real life context’ (ref. 36 p. 13), which is what we were aiming to do. Finally, case studies are thought to be suited to exploring ‘how’ and ‘why’ questions,^36^ too complex for a research design based around an experiment or survey. Thus, the use of case studies was thought to be the most appropriate research design as we were able to gather a range of data, including contextual information, by concentrating on four specific CCGs in England. We were able to pursue our research questions in depth, informed by two sets of in-depth interviews and the examination of local documents (including CCG commissioning strategies and board minutes). The documents were used to help us find out how the CCGs actually used competition and cooperation in practice and will not be reported on separately (save for the table indicating the types of services which were subject to tendering).

In the first phase of the fieldwork, between August 2013 and June 2014, we carried out 33 interviews, 13 with senior commissioners and 20 with provider managers, including independent providers, in four CCGs across England. The interviews were conducted by a team of experienced qualitative researchers (DO, ES, AC, NP and LW) who did not know the participants prior to this study. Participants were approached by email or a phone call in the first instance, were given written documentation describing the aims of this research and given an opportunity to ask questions prior to giving their consent to participate. Case study sites comprised a mix of rural and urban settings and were located in the North, Midlands and outer London (see tables 1 and 2) in order to obtain a range of experiences in respect of numbers of local providers and proximity of other CCGs, as well as different parts of England. CCG1 was located in the North of England and covered a population smaller than the average for CCGs in England. There were areas of high population density in its largest town and rural areas. There was one main acute provider, an FT. There were two main CHS providers one of which was the main acute provider and the other an NHS trust dedicated to CHS. CCG2 was located in the middle of England in a large conurbation. There were several acute providers, many of which were FTs, with the major provider accounting for just over half of total acute spend by CCG2. CHS were also provided by several trusts, as were MHS. CCG3 was located in the North of England. The diverse population included large urban conurbations through to rural areas. There was one main acute care provider, an FT. There was one main provider of MH and CHS, another FT. CCG4 was coterminous with an outer London borough. The population was served predominantly by the two acute trusts taking up the bulk of the CCG's acute spend. Owing to the density of acute provision in this part of London, local patients also used other London hospitals. There was one main NHS CHS provider and two NHS MH service providers.

**Table 1:** Interviews by case study site and phase

Case study site	Location of CCG	No. of first phase interviews	No. of follow-up interviews	Total
CCG1	Rural, North	10	2	12
CCG2	Urban, Midlands	9	2	11
CCG3	Mixed, North	7	2	9
CCG4	Outer London	7	3	10

CCG, clinical commissioning groups.

**Table 2:** Interviews by case study site and type

	CCG1	CCG2	CCG3	CCG4
Interviews with commissioners	8	5	3	5
Interviews with providers				
NHS				
Acute	2	1	2	2
Community	1	2	3	2
Mental health	0	1	0	0
Independent				
Acute	0	0	0	0
Community	1	2	1	1
Mental health	0	0	0	0

CCG, clinical commissioning groups; NHS, National Health Service.

The original purpose of the follow-up interviews in 2015 was to find out whether there were any changes in local actors’ understanding of the regulatory regime or in their use of competition over the passage of time since the first fieldwork phase. Our research design envisaged that we would interview fewer people at this stage, as we were not planning to go into such depth as in the initial phase, when we wished to investigate actors’ understanding of the new rules. The 5YFV was published in October 2014 and it was clear that this had the potential to constitute an important policy change, so we took specific note of respondents’ views of its potential and actual effects in the follow-up interviews. These interviews were almost entirely of commissioners, as we were more interested in their understanding of this policy change, as any changes in their behaviour had the potential to shape how competition and cooperation were used. (However, we took an opportunity which presented itself to interview one NHS provider).

In the second phase of data collection, between July and October 2015, we carried out nine follow-up interviews with eight commissioners (and one provider) to gauge any changes in the views and experiences. We mainly focused on commissioners to gauge the effect on them of the 5YFV.

Over the two phases, we conducted 42 interviews. The majority of the interviews were conducted face-to-face in participants’ workplaces, except in two cases where the interviews were conducted over the telephone. The interviews were conducted in private settings with no non-participants present. They lasted ∼1 hour and were audio recorded. Within the four case study sites, participants were purposively selected to include those managers most knowledgeable about either the rationales for local commissioning decisions or providers’ experiences of competition and cooperation locally. Additional interviews with appropriate managers in the relevant organisations were conducted in order to achieve data saturation. All but one of the commissioners interviewed were senior level managers. In one instance, we interviewed a former GP commissioner. The interviewed provider managers were all senior managers. They were contracting leads (in NHS providers) or chief executives (the latter often acting as the former in small independent providers).

The interviews explored commissioners’ and providers’ understanding of policy and regulations regarding the use of competition and cooperation in commissioning NHS services. We also explored their experiences of tendering and bidding for tenders, as well as collaborative working. The study had received research governance approval from each participating organisation.

Three authors (PA, DO and ES) agreed a thematic coding framework derived from the research questions, the literature on competition and cooperation and the data. The major themes covered the understanding of current policy including incentives to cooperate and compete, views on sector regulators, impact of HSCA 2012 and amount of local discretion, personal views on the role of competition and cooperation in the NHS system and specific examples of competition and cooperation in the local context including the rationale that led managers to adopt a particular mechanism, including any barriers and facilitating factors. The interviews were transcribed, uploaded to NVivo and coded (by DO and ES) using the agreed coding framework. The principal researchers (PA, DO and ES) met periodically to check whether the coding framework was working well and to agree to any necessary modifications.

We wanted to obtain comprehensive data on services which had been competitively tendered in the four case study sites to understand the extent of marketisation, and in respect of which types of services. There was no single source of such information. We used our interviews, case study sites documents, public procurement databases and email correspondence with commissioners to collect it. We were able to build an indicative list of services, which were put out to tender by the CCGs or their immediate predecessors, which is contained in table 3 below. This list needs to be treated with caution, as it may not be comprehensive.

**Table 3:** Indicative list of services tendered in the four case study sites (year of tender, where available; and value, where available)

CCG1	CCG2	CCG3	CCG4
Two aspects of Diabetic retinal screening (2009, £110–£130 K; and £350–£450 k) Dental services (2009, £2.6 m) Clinical pharmacy support (2009, £500–£700 K per annum) Lymphedema service (2009, £700–£900 K) Substance misuse (2009) Independent sector Treatment centre (2009) Community oxygen (2010) Intermediate care (2010) AQP podiatry (2012) Orthodontics (2012, £3 m) Intermediate care (2015) Diabetes service (2015)	AWP Chronic Disease Self-management Programme (2009, £200–£500 K) Continuing healthcare (2010) Urgent care transport (2010) Breastfeeding peer support (2011, £150–£180 K) Sexual Assault Referral Centre (2012, £960 K) AWP smoking cessation (2012, £600–£790 K) Pathology laboratory (2012, £1.8–£2 m) AQP Podiatry (2012) AQP Adult hearing (2012) AQP Wheelchairs (2012) Intermediate care (2013, up to £500 K) Palliative Care (2013, £36 K) Assisted conception (2013, c. £8.5 m) A nationally required new service (2014, £45–130 m, joint with other CCGs in the region) Termination of pregnancy (2014, £5.3 m) End-of-life care service Diagnostics, care and treatment Home oxygen Drug and alcohol treatment	GP-led urgent care centre (2009) Wheelchairs (2013) Community ophthalmology (2013) Midwifery (2014) AWP community endoscopy ISTC Health and social care transport (joint national) Care homes (joint regional) Urgent care transport (joint national)	Urgent care centre (2008, £500 K-£2 m) GP-led health centre (2009, £3–5 m) Drug and alcohol services (2011, £4.8 m) Primary mental Health(2011, £3.3 m) Chlamydia screening (2011, £1.8–£2.4 m) Speech and language therapy (2012, £1.8 m) Smoking cessation (2012, £1.1 m) NHS 111 pilot (2012, £880 K-£1.3 m) Clinical assessment service—telephony booking and management service (2012, c. £200 K) Health improvement (2012, c. £1.2 m) AQP MSK (2012) AQP Podiatry (2012) Rehabilitation support (2013) Ophthalmology Phlebotomy

AQP, any qualified provider; AWP, any willing provider; CCG, clinical commissioning groups; ISTC, Independent Sector Treatment Centre; GP, general practitioner; MSK, musculoskeletal service; NHS, National Health Service.

## Results

### Understanding and experience of regulatory framework under HSCA 2012

As actors’ understandings of the rules under which they operate are crucial in determining their behaviour, participants were asked about their understanding of policy, guidance and the regulatory framework regarding the use of competition and cooperation in commissioning NHS services.

Commissioners in each site considered the current policy confusing as they were expected to drive competition and integrate services, which they found to be contradictory:

Those two drivers can compete against each other. (Commissioner 3, CCG4, May 2014)

Some commissioners were awaiting guidance on how to implement policy, or commented that where there was guidance, interpretation was likened to ‘trawling through treacle’ (Commissioner 1, CCG1, May 2014). This commissioner considered that the ambiguity led to people overcomplicating policy implementation.

When asked about their understanding about whether the current policies required them to tender all services, commissioners were convinced that this was not the case. There was a consensus that although there was no mandate to tender all services, there was a requirement to justify the occasions when competitive procurement was not pursued.

We don't have to tender all services, there are exceptions. But I think the default position is that we are expected to tender services, as a generality. So we have to, I think, the expectation is that you will explain why you haven't. (Commissioner 1, CCG2, November 2013)

Lack of explicit, unambiguous guidance, about the role of competition in commissioning clinical services, could in some cases play to commissioners’ advantage by increasing their freedom. However, it also increased the freedom of providers to challenge commissioning decisions and/or to interpret the regulatory uncertainty to *their* advantage. A commissioner from CCG1 cited a case of a private provider offering maternity services in the region and expecting to be paid by the local CCGs despite not being commissioned by the CCGs. Such provider behaviour, driven by patient choice and effectively bypassing commissioners, undermined the level of control commissioners had over their local health economies. In general, the interviewee remarked that often commissioners’ strategy was to comply with the national framework but immediately find ways round it to adapt to the local circumstances (Commissioner 1, CCG2, November 2013).

Provider managers were also confused about the meaning of the competition rules. They echoed commissioners’ concerns about the vagueness and complexity of the formal rules and a need for better guidance.

The lack of sufficiently specific guidance about the HSCA 2012 resulted in commissioners having to consult sector regulators. Commissioners were often quite critical of the role of sector regulators, mainly due to their alleged inability to provide clear guidance in particular cases. One commissioner expressed the view that sector regulators (as interpreters of national policy) could not decide whether the priorities lay with increasing competition or with fostering integration among providers. Such a view was a consequence in part of the decision to reject a merger proposal between the Royal Bournemouth and Christchurch Hospitals NHS Foundation Trust and Poole Hospital NHS Foundation Trust hospitals taken in October 2013 by the CC, which sent shock waves through commissioning world.

You know, the debacle of Poole and Bournemouth, crikey. You know, 10 years ago that merger would have just happened. They'd have done a public consultation and whatever, but it would have just happened because it was the right thing from a quality patient side of things and the right thing from a commercial viability, and recognising really in Poole and Bournemouth, (…), there's no choice. (Commissioner 3, CCG1, April 2014)

Another CCG1 commissioner expressed their disappointment over the lack of clear guidance from Monitor after the CCG came across the private maternity provider billing them for activity which had not been contracted (as mentioned above). Monitor's stance was seen as ‘very wishy-washy’ and their interventions were not ‘effective’ or ‘timely’ (Commissioner 4, CCG1, April 2014).

A further complex issue on which the CCGs wanted to consult the regulators related to opening up services to competition when these services were not a commissioning priority. Commissioners from CCG1 were approached by potential providers (in this case a GP practice) wishing to provide CHS when the commissioners were not prioritising this issue. (In fact, the question of a GP practice wanting to provide CHS was problematic for other reasons: CCGs are GP membership organisations, giving rise to potential conflicts of interest due to GPs’ dual role as commissioners and providers of services.) In another case, a commissioner from CCG3 was concerned that Monitor might intervene and put a stop to the ‘controlled market approach’ which CCG3 pursued (Commissioner 1, CCG3, August 2013). The worry remained that if one were forced to follow a strict interpretation of the rules, decisions such as not putting some services out to tender, would not be allowed.

In common with commissioners, the regulation of the health sector was seen as muddled by provider managers, with Monitor having conflicting duties. One felt that the relationship between the different regulators was not clear and there was no overall organisation responsible for regulation:

The interactions between the different regulators is confused. No, there used to be … an organisation that was clearly responsible for … holding the ring, in the shape of SHAs [Strategic Health Authorities], that's disappeared. (Provider 2, NHS, acute, CCG2, March 2014)

An independent provider manager noted that Monitor did not show much interest in the place of small providers in the NHS market, and was focusing its attention on large NHS acute hospitals.

### Changes in understanding of the regulatory framework after the publication of The Five Year Forward View

Commissioners in all the case study sites, who were reinterviewed in 2015, after the publication of the 5YFV, noted a change in tone of national policy messages towards greater promotion of collaboration in commissioning. They thought that the 5YFV legitimised local cooperative initiatives aiming to transform services and it allowed them greater latitude in deciding whether to tender out services. However, at the same time, commissioners pointed out that none of the underlying rules guiding procurement of clinical services had changed as a result of the 5YFV and warned that the rules could not be disregarded completely.

I think over the last 12 months, it's almost as an unwritten rule there does seem to be a relaxing of the rules around the need to go to full procurement. If you can demonstrate it's in the best interests of the patient, you can stick with your local patch and it is seen to be almost an unwritten rule, and if you ask me to go and find a document that says this is how you should do, I'd struggle, but it's how everyone seems to be operating now. (Commissioner 1, CCG3, July 2015)

The different national bodies, some in favour of cooperation (NHS England) and some still promoting the use of competition (Monitor) were sending conflicting messages. Most of the reinterviewed commissioners saw a need for competition to remain available to them as a commissioning tool to use at their discretion.

The follow-up interview we conducted with one of the providers in 2015 highlighted many important changes in the way providers perceived the rules in respect of competition and cooperation in commissioning of the NHS services compared with 2013/14. This interviewee noted that the publication of the 5YFV did not change his understanding of the policies and rules about the use of competition in commissioning. However, it had encouraged, and to an extent legitimised, collaborative ways of working. Different providers, and the CCG4 CHS trust in particular, had been increasingly engaging in testing the rules by getting together and working more collaboratively, pushing ‘the art of the possible’ (Provider 5, NHS, community and MH, CCG 4 October 2015). Such grassroots local developments driven by the genuine pressing concerns over costs and quality of services tended to override strict interpretations of the legal framework that so far remained unchanged.

### Use and experience of competition and cooperative mechanisms

In order to understand how competition and cooperation were used locally in our case study sites, we investigated the major service delivery issues, so that we could see how they were approached.

The urgent need to find savings had been made clear by NHS England in July 2013 and dominated local agendas. Each case study site was engaged in efforts to move care out of hospital into the community, in the hope that money would be saved.

The game-changer is going to be the Call to Action, 30 billion [funding gap]. There's got to be major strategic change of hospital services and delivering care in the community, sustaining people and people sustaining themselves through self-care. We just haven't got the money and we're not going to. (Commissioner 1, CCG4, November 2013)

In order to tackle these big service reconfiguration challenges, the four CCGs were generally exploring collaborative approaches. Commissioners on the whole took the route of coordinating cooperation between themselves and providers. They did not use competitive processes to make major changes. The complexity of the issues involved in such reconfigurations required an iterative approach using a series of meetings with local providers at which actors were encouraged to come to a collective agreement about how changes would be made.

Commissioners in all sites were exploring the option of using outcome-based commissioning approaches as well as lead provider models for a range of different services. In two sites—CCG4 and CCG1—the outcome-based commissioning approach was going to be used to redesign the provision of CHS. Commissioners hoped that prime provider models and outcome-based contracts would remove cost pressures associated with the ‘open chequebook’ (Commissioner 3, CCG1, April 2014) pricing structure of PbR by moving to capitated budgets. These new organisational models were likely to reduce the amount of local choice available to patients by the formation of larger organisations and the reduction in the number of possible organisations to choose from. Although these approaches could have involved competitive tendering, they did not do so in our case study sites. Instead, providers were encouraged to stop treating themselves as ‘little entities’ in a competitive game looking after their own interests and to start to acknowledge that lack of money in the local health system required a change of mentality (as well as more efficient processes). The way our case study sites embarked on tackling such challenges was by talking with existing providers, gathering intelligence and data needed for service reviews, assessing the performance of services and areas that required change and finally looking for contractual levers to use to deliver the change. Going to the open market was seen as an option of last resort in respect of big service delivery transformations, and collaborative methods were preferred.

Although competition was not used to bring about large-scale service reconfigurations, commissioners in our case study sites did use competition for the market, using competitive tendering, in respect of smaller services. Table 3 contains an indicative list of services, which were put out to competitive tender in the four CCGs or their PCT predecessors. It shows that the services, which were put out to tender, were mostly primary care, community and diagnostic services. Individual services delivered in acute hospital settings were not often tendered, and no services in a whole hospital had been subject to competitive tendering. Commissioners in CCG3 indicated that their local health economy culture was a collaborative one, and that they did not wish to use competitive methods, if they could be avoided.

We asked commissioners to tell us about their experiences of tendering out a service, once they had decided on this route. Some commissioners stressed the importance of testing the market prior to issuing a formal invitation to tender. This was seen as necessary to ascertain provider interest in order to avoid the situation of having to go through a costly tendering process to end up with the same or a worse quality provider. Hosting provider engagement events was seen as a way to gauge provider interest and served as a general tool for mobilising existing providers to improve their performance. The use of adverts and market engagement events was a warning sign for incumbent providers that they ought to take commissioners seriously.

The decision to procure something in terms of any service, no matter what it might be, does sort of stimulate the market and people then start to look at—to you as an organisation, knowing that, you know, you use—you're using a procurement route more than you won't, in terms of delivering services. (Commissioner 2, CCG2, December 2013)

When assessing the bids, commissioners applied a number of criteria to assess the suitability of potential providers. In particular, they looked at whether providers were financially sustainable and capable of delivering the service.

Quality, value for money, safety, governance. What else would you look for? Whether the capabilities of whether somebody's big enough to deliver it, if it's big? If we're talking about the community services, if it was are they big enough? Have they got the track record? So have they got expertise in this area? (Commissioner 4, CCG1, April 2014)

Commissioners from CCG4 stated that they would have made more use of competitive tendering to procure services but felt constrained by the lack of resources to run such processes. One commissioner noted that one would need ‘a whole army of people’ to use competitive procurement on a large scale (Commissioner 3, CCG4, May 2014).

I would be more aggressive and probably more prone to tendering if I had more resource devoted to it and I would be moving resource from serious redesign or basic commissioning function into procuring and doing things in a more structured market interventionist way. (Commissioner 1, CCG4, November 2013)

Most commissioners noted how time consuming and expensive tendering was. The CCG4 commissioner reflected on the high costs of the primary care MHS procurement.

We did it in-house led by the Commissioning Manager for Mental Health, but we had two Project Managers, you know, it wasn't cheap to do this. And in total, we probably—I think we spent a quarter of a million pounds doing the specification, the involvement and then the procurement. You know, most of it was on salaries for the Project Managers and legal cost in terms of getting expert advice from [legal firm], to make sure we ran the process. You've got to get it right, because you could be challenged. [The incumbent provider] tried to challenge us and we had to send them all the documentation. (Commissioner 1, CCG4, November 2013)

We also asked providers about their experiences of participating in a tendering exercise. They reported that tendering was very resource intensive for them, and that it was made more difficult by the lack of experience of many commissioners. This affected the efficiency of the processes. Moreover, several providers were concerned that commissioners were relying on them to provide information required to write the tender specifications, rather than being able to do this themselves. Many providers noted that commissioners were still not able to specify the service at later stages of the process, and relied on each provider tendering to give details about what the service should comprise.

Furthermore, market engagement exercises were described as a ‘courting process’ which was difficult to manage from the incumbent provider's point of view. The provider was trying to convince the commissioner that tendering the service was not necessary, but at the same time trying to remain part of the tendering process. It was a difficult balancing act for an acute provider, which wanted to maintain their chances of winning the bid, knowing that at the same they might disclose something that would feed into the service specification and work to their disadvantage.

The level of trust that, you know, how open you are and how much you're giving to a specification because if you give too much, they use that for the specification that someone else can then win for. So it's quite a difficult one. (Provider 4, NHS, acute, CCG3, December 2013)

Once the formal tendering process was underway, it was characterised by one provider as ‘chaotic’ due to the short notice periods for submitting the paperwork. A common complaint was the large amount of resources that NHS providers had to dedicate to pursue competitive tenders.

We lose sight of while every provider's bidding for this work the resource involved to answer them and particularly the stages depending on what process they use is substantial. So, you know, you've got a pre-qualification questionnaire, then you have an invitation to tender, then you have a presentation, then you've got mobilisation. And the amount of resource and financial input into them is significant and you question how much that benefits the patient. The outcome possibly does but if you've had seven organisations doing that and only one is successful. (Provider 4, NHS, acute, CCG3, December 2013)

In fact, some providers could not afford to bid, due to their poor financial situations.

I think the challenges are making sure that you have sufficient money to invest in bidding for things, because it takes a lot of time and effort, particularly from an Operational Team, a Clinical Team, but also the finance and information you support. And traditionally, you know, ‘cause we don't have any money, we're poor, we don't have a budget set aside, so you're asking people to do it on top of their normal job. (Provider 4, NHS, acute, CCG4, April 2014)

Often the information released by commissioners was not sufficient for a provider to make a cost benefit assessment before deciding whether the tender would be viable.

We've recently bid for a [XX] service in [locality X], now we made a tactical decision in doing that to actually massively inflate the costs, the price, because the case mix information on the service was none existent, so they told us the kind of service they want, they couldn't tell us how many of what, well, I mean, again, it's just…as far as I'm concerned, it's appalling! (Provider 5, NHS, acute, CCG3, December 2013)

As part of our research into how commissioners were using competition in the NHS, we investigated whether CCGs had a strategy to develop the market for non-NHS independent providers. This is an interesting question because it indicates the extent to which commissioners saw competition as a method of increasing diversity of supply. None of the sites had a deliberate strategy to enable greater involvement by for-profit providers in the provision of services. Instead, they appeared as contractors to the NHS on an ad hoc basis.

We've used the independent sector to develop services that are substitutional for secondary care, or to outplace secondary care services. We've used them equally for some aspects of what's traditionally been community provision. So it's case by case. I don't think we have a—there isn't a kind of an overarching policy imperative to either grow or restrict the private sector. We use it where it's relevant. (Commissioner 1, CCG2, Nov 2013)

Some commissioners were at pains to point out that they were careful to treat all providers equally, irrespective of whether they were NHS or not. One CCG (CCG4) was more enthusiastic about their entry into the NHS quasi-market than the others. On the other hand, all of the CCGs were actively engaged in supporting local third sector organisations. Providers did not agree that they were all treated equally, and, in particular, some independent providers complained about being at a disadvantage. It appeared that, on the whole small and medium size independent providers did not experience the same level of communication and engagement from commissioners as larger independent providers or NHS trusts.

The two [NHS] trusts are kept informed but we're just treated terribly, I would say. We're not kept informed with things, although they do come to us often if they need—for instance, the hospital were under pressure, winter pressures, and they didn't have enough doctors. The commissioner came and said, I wonder, could you put some of your GPs in there just for the next fortnight, just to take some of the pressure off A&E? They'll do that all the time but they won't keep us informed about anything else that's going on around our contract, et cetera. (Provider 6, independent, CCG3, March 2014).

Apart from a specific local commissioning climate created by their main commissioning CCG (eg, in CCG3, where the commissioners did not favour the use of competition), the different types of providers (acute, CHS and MHS) faced different constraining factors. Many of these constraints were determined at the national level, importantly, different pricing mechanisms for different types of services. Furthermore, owing to their different sizes, and types of services being delivered, the market horizon of some providers stretched beyond one particular CCG area to other CCGs and to markets created by different types of commissioning bodies, for example, local authorities. Thus, the position of a particular provider within the NHS was a product of the number of factors such as the nature of the local market, the type of services they provided and whether they were able to reach to other markets.

Providers in our sites had a first-hand experience of how the principles of competition and cooperation worked together in healthcare delivery practice. Providers had to comply with dual incentives to cooperate and compete with other providers in the system and, importantly, to engage with commissioners in service planning. This led to circumstances in which providers sometimes collaborated with their competitors and competed with their collaborators. This applied to all types of providers, including independents.

Providers expressed concern about barriers preventing fair competition. One important issue was the different pricing mechanisms for acute and out of hospital care. An interviewee contrasted the position of MHS (which had block contracts, effectively a fixed budget) with that of the acute trusts, which could expand their provision due to being paid on a cost per case basis through PbR.

A third of our Trust is Specialists, mostly Secure Services. But we can't say, “We're fantastic at Secure Services, we'll take five more patients,” and get paid for it, because we're just on a block [contract]. So we've got no way of flexing up on services that we're good on. If you work in [local acute trust], big, gleaming, enormous university hospital, and they decide, “Actually, we've got some real Specialists in trauma,” and the Air Ambulance starts flying people in from [locality X] who have trauma, they may not get exactly the money that they want, but no one turns round to them and says, “We can't pay you for that”. (Provider 1, NHS, CHS and MH, CCG2, April 2014)

Whether competing or cooperating in respect of gaining (or trying not to lose) patients, all providers noted that they cooperated at the clinical level, to ensure that patients moved smoothly between organisations. Other reasons for collaborating included partnerships to deliver non-clinical services, participating in local service transformation programmes and forming partnerships to compete together for tenders.

Many interviewees distinguished between being ‘forced’ to compete or ‘forced’ to cooperate by different parts of NHS organisational hierarchy and choosing to compete or cooperate because of the provider's internal decision-making. Top-down ‘enforced’ competition seemed to have affected CCG commissioners and providers and was viewed as counterproductive.

They tried tendering Pathology, but that was an interesting example because that was being forced by the old Strategic Projects Team in [one region] Health Authority, and it was—it's fascinating as an exercise. (…) They tried imposing the same sort of model in the [region of CCG2], and so long as an SHA was there to wave the big stick, the PCTs played along with it. And 6 months after the SHAs were abolished, the CCGs said, “No, we're not going to do that anymore. We never wanted to do it in the first place. You were forced—you forced us into it.” There's a huge degree of inertia in all parts of the system. (Provider 2, NHS, acute, CCG2, March 2014)

In addition to understanding how providers competed and cooperated with each other in our case study sites, it was important to understand providers’ perceptions of their relationships with the commissioners. We found that, in different circumstances, providers experienced threats of competition and encouragement to collaborate with the commissioners and each other. They took the view that both of these techniques were being used by commissioners to influence their behaviour, and thus improve services. Providers of services out of hospital were more susceptible to threats of tendering than acute providers. CHS and MHS providers thought that the balance of power rested firmly with the commissioners. This is because CHS were more likely to be put out to tender and because block contract payments prevented expansion of services on providers’ initiative.

The commissioner has all the power here because they can tender. And if I don't win the tender, I lose it. (Provider 1, NHS, community and/MH, CCG2, April 2014)

However, all types of providers could see the advantages of being encouraged to collaborate, and of having good working relationships with their commissioners. These might even obviate the need for commissioners to put services out to tender.

### Changes in behaviour following the publication of The Five Year Forward View

The 5YFV (2014) emphasised the need for NHS organisations to cooperate with each other, and did not appear to favour the use of competition as a mechanism to improve services.

The follow-up interviews conducted in the latter part of 2015 painted a mixed picture of commissioning practices in the four case study sites. On the one hand, all the case study sites were undertaking major local service transformation using collaborative working with providers (and had been doing so before the 5YFV was published). Interviewed in 2015, the CCG2 commissioner stated that collaboration was their default approach, ‘a kind of way of life’ which was ‘punctuated by odd moments where I have to formalise things…on occasion into contractual or permanent or competitive relationships’ (Commissioner 1, CCG2, July 2015).

On the other hand, three out of four sites (apart from CCG4) were using competitive tendering to procure some smaller, more peripheral services. CCG4 did not rule out doing so in the future. This use of competition included CCG3, which had not undertaken any competitive procurement on its own in 2013/2014, and had been the least keen to do so at that time. By 2015 CCG3 had also been using a full procurement route for commissioning some of the services which experienced ‘poor quality, poor outcomes’ issues (Commissioner 1, CCG3, July 2015) or in order to increase patient choice.

It was the first time we've ever done this within [CCG3 area], a full procurement. So it went out to tender and a preferred provider was appointed, not [the local acute provider], although to be fair to [the local acute provider], they've now come to the table and are working with the new provider, but as a result of that, we have created a much better service for the patients because it's more locally focused. It's not hospital focused. (Commissioner 1, CCG3, July 2015)

This CCG3 commissioner had found the competitive approach useful in this case as it injected new ideas into a ‘very conservative’ local landscape*.* (Commissioner 1, CCG3, July 2015). Another CCG3 commissioner did not exclude the possibility of competitive tendering being used in future commissioning (Commissioner 2, CCG3, October 2015).

Although noticing shifts in policy rhetoric towards cooperation following the 5YFV, in their day-to-day practices, commissioners were exercising their own judgement and making use of all tools available to them. In fact, taking account of CCG3, we can see that, over time, a wider range of commissioning mechanisms were being used.

Competition was being used alongside work on various projects to integrate services. However, the problem was how to integrate different integration work streams under one strategy.

You've got to understand how many bids there are going on, and that's the confusing thing. We're a pioneer. [Acute trust] are a vanguard for the care homes. I believe they've got a bid in as well with a group of other providers around something else, and there's so many things going off at the moment it's hard to keep a track. (Commissioner 1, CCG1, August 2015)

## Discussion and conclusions

The study has certain limitations. *First*, as the study design consisted of four in-depth case studies, it is not possible to generalise to the whole NHS. In particular, it should be noted that none of the CCGs in our study had decided to use competitive tendering to achieve major service reconfiguration but national information (mainly reported by the *Health Service Journal*) indicates that some CCGs had done so, although with variable results. (For example, see the history of the NHS owned Hinchingbrooke Hospital, the whole of which was put out to tender and run by a for profit company, Circle. By 2015, Circle had withdrawn from the franchise contract because it was unable to achieve the financial savings it had promised the NHS, and the hospital's services had been subject to critical inspection reports by the Care Quality Commission (CQC). On the other hand, some major tenders appear to have been more successful. For example, in 2014 Circle was awarded a 5-year £120 million musculoskeletal (MSK) contract by Bedfordshire CCG and a consortium of NHS and independent providers won a £210 million MSK contract in Sussex.)

*Second*, it was not possible to find the full details of the tenders undertaken in our four case study sites, due to the very poor quality of routine data available. This problem affects the whole of the English NHS and means that one cannot ascertain with any certainty the extent and nature of the use of competitive commissioning across the NHS, nor the extent of market entry by independent providers. The possibility of generating evidence about the NHS is compromised, and thus the ability to make evidence-based policies.

*Finally*, it should be noted that we did not collect comparative data about the amount of resources available to be spent on procurement in each case study CCG. We were therefore unable to speculate about whether there were differences which might have affected the CCGs’ behaviour, in addition to other factors such as local market structure and attitudes of senior commissioning staff.

Our findings concerning the lack of clarity of the regulatory regime for local actors are important. As Ostrom points out, actors need to understand the rules of the game in order to know how to relate to each other, and these rules are vital in setting the context and limits within which local actors can operate.^35^ As we have shown, the interpretation of these rules by regulators, national authorities and local actors were not clear to participants and changed over time. This finding supports those of Krachler and Greer.^34^ It remains government policy (as well as being enshrined in the European procurement regulations), that there should be a ‘fair playing field’ for all providers of care to NHS patients in order to enable the quasi-market to operate effectively, with the aim of producing efficient high-quality care.^37^ One prerequisite for such a ‘fair playing field’ is that all actors understand the rules governing that market. It is also important that commissioners actually treat all providers equally, which our study indicated they were not doing at all times (as Krachler and Greer^34^ also found).

It is not surprising that commissioners and providers used a judicious mixture of competition and cooperation in their dealings with each other. This behaviour is common in most markets for complex goods and services.^38^ ^39^ One of the important reasons for doing so is to reduce transaction costs, as our participants explained. This links directly to the arguments made by eminent institutional economists^23^ ^24^—there are certain goods and services whose characteristics indicate that non-market institutional structures will be more efficient than using markets, due to the transaction costs incurred in operating such markets.

Furthermore, the influence of the ‘institutional logics’ identified by Tuohy are also important to understanding how the NHS operates.^3^ The NHS has a long history of hierarchical modes of control, which is difficult to change in a short period of time. Previous research on the NHS has indicated that the earlier versions of the quasi-market introduced by the Conservatives in the 1990s and New Labour from 2001 onwards had not blunted the dominance of hierarchical methods of coordination despite the introduction of market-like structures. Checkland *et al*,^40^ using theories of new institutionalism, identified a lack of fit between the norms permeating the NHS such as the focus on individual patients and seeing the NHS as a common enterprise and the formal rules of commissioning pushing for greater marketisation of relationships between different actors within the health system under the New Labour version of the quasi-market.^41^ That study suggested that hybridity instigated by marketisation of the NHS was present at that time only at the level of structures, while norms governing actors’ behaviour remained relatively unaffected. Our study has shown that this is substantially true in respect of the third incarnation of the NHS quasi-market under the HSCA 2012. However, it is important to note that this is not the whole story. Checkland *et al*^40^ point out that institutional change may occur in future if commissioning principles become more embedded in the NHS culture. Our study shows that there are signs that competitive forces are gradually taking hold in respect of some more marginal services, and especially in respect of CHS and MHS. It appears that it is possible for NHS norms and culture to change.^42^

The implications of our study for policymakers are several. Local commissioners should be allowed to make their own decisions about which modes of commissioning are most appropriate in their particular circumstances, and in respect of particular services. Setting up nationally imposed rules about what mechanisms must be used is unhelpful (and probably will not be adhered to). It appears that in most circumstances, the use of cooperative modes of coordination is likely to be more appropriate. Fortunately, the recent policy developments under the 5YFV indicate this is the direction of travel. At the same time, it is important to clarify the rules of the game for local actors. It may be politically unpalatable, but the regulatory framework of the HSCA 2012 needs revisiting to ensure that commissioners have a clear choice about whether to use competitive mechanisms or not.
